# Pilot biomechanical study of complex upper-limb movements in patients with RSA using inertial sensors: Feasibility of sport-specific gestures

**DOI:** 10.1177/17585732261419033

**Published:** 2026-02-10

**Authors:** Giorgio Ippolito, Marco Damo, Sergio Ferraro, Dante Trabassi, Mariano Serrao, Riccardo Maria Lanzetti, Michele Francesco Surace, Daniele Mazza

**Affiliations:** 1 Trauma & Orthopaedic Department, Icot Hospital “Marco Pasquali”, Latina, Italy; 2 Department of Orthopedics and Traumatology, Asl1 SSR Liguria, Sanremo, Italy; 3 Department of Medico-Surgical Sciences and Biotechnologies, 9311 Sapienza University of Rome, Rome, Italy; 4 18656 Orthopaedics and Traumatology Unit, Department of Emergeny and Acceptance, Azienda Ospedialiera San Camillo-Forlanini, Rome, Italy; 5 Department of Biotechnology and Sciences for Life “Insubria” University, 226462 Varese, Italy; 6 Department of Orthopedics and Traumatology, 486464 Istituto Dermopatico dell’Immacolata, Rome, Italy

**Keywords:** Return to sport, reverse shoulder arthroplasty, shoulder surgery, shoulder biomechanics

## Abstract

**Background:**

This study aimed to evaluate recovery of complex upper-limb movements from a kinematic and biomechanical perspective in patients undergoing Reverse Total Shoulder Arthroplasty (RSA), comparing movement quality during athletic gestures with healthy controls.

**Methods:**

Two groups were analyzed: patients with RSA and healthy individuals without shoulder pathology. Participants performed basic shoulder tasks (flexion–extension and abduction–adduction) and three athletic gestures of increasing complexity: boccia throw, golf swing, and padel víbora stroke. Kinematic data (joint angles, angular velocities, and accelerations) were collected using a wearable inertial motion analysis system (Movit System G1).

**Results:**

Controls demonstrated a greater range of motion (maximum joint angle: 184.0° vs. 144.03°), though differences were not statistically significant. Angular velocities and accelerations were largely comparable between groups, indicating that patients with RSA adopt conservative yet functional movement strategies. No significant differences were observed during the boccia throw or golf swing. The víbora stroke showed the highest variability but remained within functional limits in both groups.

**Conclusions:**

This pilot feasibility study suggests that patients with RSA can perform complex upper-limb and sport-specific movements with biomechanical patterns comparable to healthy individuals. Although limited by small sample size, large effect sizes indicate clinically relevant differences, supporting the need for larger, confirmatory studies.

## Introduction

Reverse Total Shoulder Arthroplasty (RSA) has become an increasingly adopted surgical solution for managing advanced glenohumeral osteoarthritis and irreparable rotator cuff tears, especially in elderly or low-demand patients. Originally conceived as a salvage procedure for pseudoparalysis, RSA has demonstrated significant improvements in pain relief, functional recovery, and overall patient satisfaction.^1^ Over the past decade, the indications for RSA have broadened, extending to younger, more active individuals, including those wishing to return to sports (RTSs) or physically demanding occupations.^2,3^

In parallel with this demographic shift, there has been growing interest in evaluating functional outcomes beyond standard clinical measures, such as range of motion (ROM) and pain scores. RTS is now recognized as an important patient-reported outcome measure (PROM), particularly for patients under 70 years of age.^4^ Studies have reported RTS rates between 50% and 80% following RSA, with many patients resuming low- to moderate-impact sports such as swimming, golf, or cycling.^5–7^ However, most of these investigations rely on self-reported surveys or basic clinical testing and do not objectively assess the biomechanical quality of movement.

Functional recovery after RSA is influenced by multiple factors, including deltoid compensation, scapulothoracic rhythm, prosthetic design (e.g. medialized vs. lateralized center of rotation), and soft tissue balance.^8^ While some studies suggest that lateralized designs offer better rotational strength and more physiological joint mechanics, the actual performance of complex, sport-specific movements has not been biomechanically validated.^9^

Currently, there is a notable gap in the literature regarding the objective kinematic assessment of patients with RSA during dynamic tasks. Most biomechanical studies have focused on daily activities of living (ADL), such as reaching or lifting, rather than high-demand athletic movements requiring speed, coordination, and multiplanar joint control.^10^ Moreover, no studies to date have explored shoulder kinematics in patients with RSA using wearable motion capture systems during sport-related tasks—a method that could provide valuable insight into compensatory strategies and functional capacity.

The present study aims to address this gap by analyzing upper-limb kinematics during both basic and complex movements in patients with RSA compared to healthy controls. Using a wearable wireless motion capture system, we assessed standard shoulder tasks (flexion-extension and abduction–adduction) as well as three sport-specific gestures of increasing complexity: the boccia throw, the golf swing, and the víbora stroke in padel.

Authors hypothesized that, despite reduced ROM, patients with RSA would demonstrate functional compensations enabling them to execute athletic tasks with movement quality comparable to healthy individuals in terms of angular velocity and acceleration, though with altered joint control and coordination.

## Materials and methods

This study involved two groups of participants. The prosthetic group included individuals who had undergone RSA with a lateral-medial prosthetic design. The control group consisted of healthy participants with no history of shoulder pathology, musculoskeletal impairment, or previous upper-limb surgery.

Participants in the prosthetic group were eligible for inclusion if they had undergone lateral-medial RSA at least 12 months prior to testing, had completed postoperative rehabilitation, and were free from complications such as infection, instability, or neurological deficits affecting the upper limb. All participants had to be capable of performing basic arm movements independently and report no pain at rest or during light activity. Exclusion criteria included any prior surgery or trauma to the contralateral shoulder, neurological or systemic musculoskeletal disorders, or inability to perform the motion tasks due to discomfort or movement limitation. In the control group, individuals were excluded if they had a history of shoulder pain, injury, or regular participation in overhead sports that could influence shoulder kinematics.

Each participant was instructed to perform a set of upper-limb motor tasks designed to evaluate shoulder mobility and function. These included fundamental joint actions such as shoulder flexion-extension and shoulder abduction–adduction, which served as baseline reference movements for comparison.

In addition to these basic tasks, three sport-specific gestures were selected for their increasing complexity and biomechanical demand: the boccia throw, the golf swing, and the víbora stroke in padel. These movements were chosen to represent a progression in the level of coordination, joint ROM, and neuromuscular control required for execution.

All movements were recorded using a wearable wireless motion capture system (Movit System G1, Captiks, Italy). The system consists of inertial measurement units (IMUs) strategically placed on anatomical landmarks of the upper limb and trunk to capture three-dimensional joint motion. Participants were allowed familiarization trials before recording to ensure consistent and confident execution of the tasks.

Data were acquired in real time under standardized testing conditions and processed using the manufacturer's software suite. For each movement, joint angles, angular velocities, and accelerations were extracted and averaged across multiple trials to account for variability and ensure reliability.

All subjects gave their informed consent for inclusion before they participated in the study. This study was conducted in accordance with the Declaration of Helsinki, and its design was approved by the Ethics Committee/Scientific Council of the ICOT Marco Pasquali Hospital in Latina, Italy (protocol no. 1; November 2024).

### Surgical technique

All patients included in the RSA group had a confirmed diagnosis of cuff tear arthropathy. Patients with proximal humeral fractures amenable to RSA, a history of joint infection, or inflammatory arthropathies such as rheumatoid arthritis were excluded from the study.

All procedures were performed by the same senior orthopedic surgeon to ensure technical consistency. A standard deltopectoral approach was used in each case, with careful dissection to protect neurovascular structures and preserve deltoid integrity. Particular attention was paid to avoiding detachment of the deltoid origin throughout the procedure.

Each patient received the same implant model: Tornier Perform^®^ Reverse Shoulder Arthroplasty (STRYKER, USA). The prosthesis design featured a lateralized glenoid component combined with a medialized humeral stem, aiming to optimize deltoid tension and joint stability. At the conclusion of the procedure, the subscapularis tendon was repaired via transosseous sutures whenever feasible.

Postoperative care followed a standardized rehabilitation protocol, implemented across all patients at the same specialized rehabilitation center. Patients were immobilized in a shoulder brace with 15° of abduction for the first 4 weeks. During this phase, gravity-assisted pendulum exercises were initiated starting on postoperative day 15. External rotation was restricted during this period to protect the repair.

Between weeks 4 and 8, patients began passive ROM exercises under the supervision of a physiotherapist. Active ROM exercises were gradually introduced over the following 6 weeks to promote neuromuscular re-education and controlled shoulder activation. After 8 weeks, patients transitioned to a home-based rehabilitation program focused on mobility maintenance and strength recovery.

This unified surgical and rehabilitation strategy ensured consistent perioperative management across all patients enrolled in the prosthetic group.

### Movit System

The Movit System G1 (Captiks, Italy) is a wearable IMU-based motion capture system validated for upper-limb kinematic studies. To ensure the validity of our methodology—especially in comparing nonhealthy individuals (patients with RSA) to healthy controls—we first established the system's performance in healthy participants. The system has previously demonstrated a mean root mean square error (RMSE) of < 3.5° in sagittal plane motion when benchmarked against gold-standard optical systems (e.g. Vicon), and correlation coefficients > 0.97, particularly for shoulder flexion-extension and abduction–adduction. These values are in line with the reported thresholds for acceptable biomechanical measurement error in both clinical and sports science applications.

Similar to studies validating IMUs for joint biomechanics in pathological populations, such as those by Arami et al.^11^^©^ and He et al.,^12^ we ensured that all testing in this study included sensor calibration trials on healthy individuals to define baseline system variability. Arami et al.^11^ reported an RMSE < 5° and intraclass correlation coefficient > 0.95 in gait and single-plane ROM using a wearable system, while He et al.^12^ demonstrated angular deviations < 4.5° in hip movement analysis, confirming the applicability of IMUs for capturing joint motion in clinical populations. In our study, all sensor placements and calibrations were standardized to minimize noise and intersubject variability. The comparative analysis was structured to detect motion deviations in patients with RSA exceeding the expected error range, supporting the interpretation of functional differences rather than sensor limitations.

Kinematic data were collected using the Movit System G1 (Captiks, Italy), a validated wireless inertial motion capture system designed for real-time biomechanical analysis. The system consists of wearable IMUs and a USB-connected receiver that communicates wirelessly via a proprietary 2.4 GHz protocol (IEEE 802.15.4 MAC), allowing simultaneous data acquisition from up to 16 sensors at a sampling rate of 100 Hz. Each sensor measures 48 × 39 × 18 mm, weighs 40 g, and integrates a 3-axis accelerometer and 3-axis gyroscope with configurable sensitivity ranges (±2 g to ±16 g and ±250°/s to ±2000°/s, respectively). Sensors are powered by rechargeable Li-Po batteries, enabling up to 6 hours of continuous use, and are managed by a 40 MHz AT32UC3A4256 microcontroller (Atmel, USA) that supports real-time data transmission. The system tracks six degrees of freedom (6-DoF) orientation using quaternion-based sensor fusion algorithms for accurate reconstruction of joint motion. Calibration follows a two-step process: first, a reference frame is established through two perpendicular 90° rotations using a calibration base; second, a T-pose alignment (arms abducted to 90°) aligns sensor axes with anatomical planes. Sensors are attached using elastic bands and plastic docking systems with Velcro to ensure stability and minimize motion artifacts across different body types. The Movit System has been validated against optical motion capture systems (e.g. Vicon), showing RMSE < 3.5° for sagittal plane angles, Pearson correlation coefficients > 0.97, and spatiotemporal gait parameter errors < 5%, confirming its high accuracy and reliability. Data acquisition was performed using Motion Studio, while postprocessing was conducted in Motion Analyzer, which provides synchronized video, three-dimensional (3D) visualization, and automated joint kinematic reporting.

### Data acquisition and signal processing

**Figure 1.**
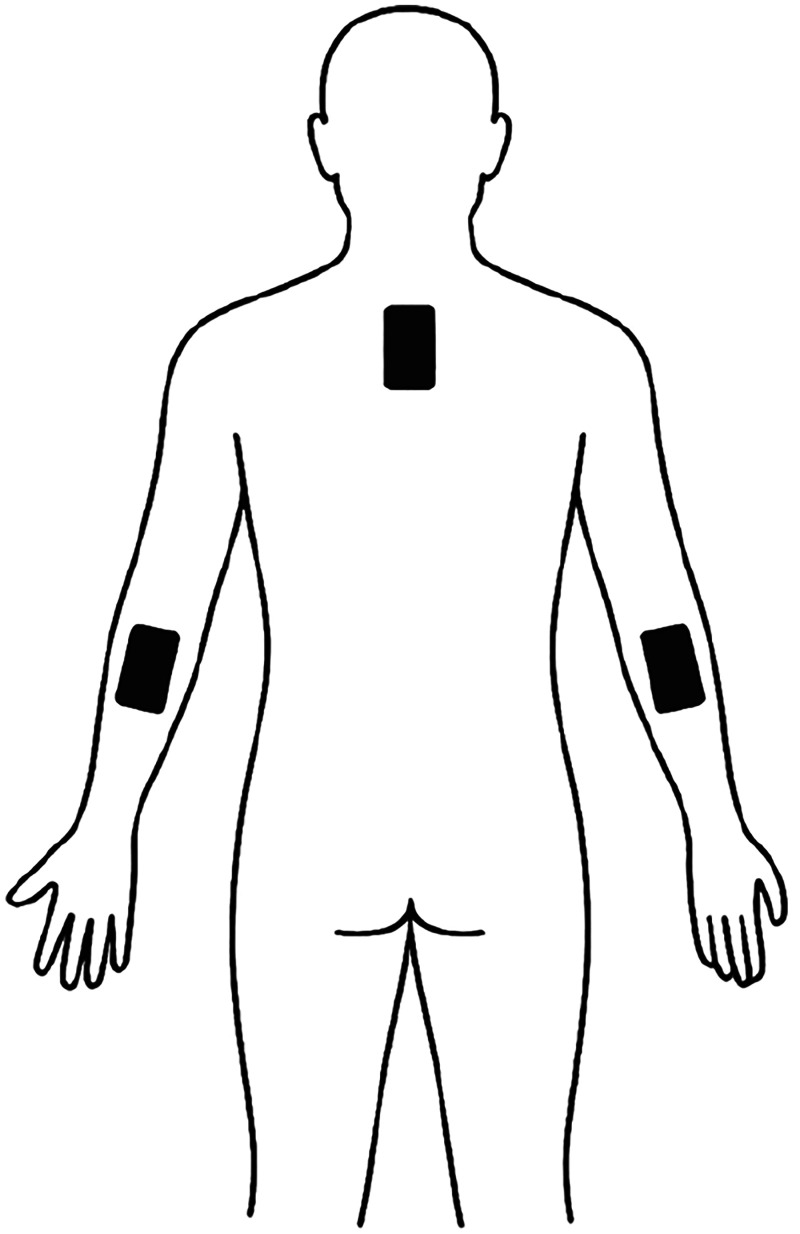
Placement of IMU sensors for trunk and upper limb kinematic analysis.

In this study, three inertial sensors from the Movit System G1 were used to capture upper-limb kinematics during task performance. Sensor placement was standardized across participants: one sensor was positioned centrally over the thoracic spine between the scapulae to monitor trunk motion and compensatory strategies, while the remaining two sensors were placed on the distal forearms, one on the prosthetic limb and the other on the contralateral side, for bilateral comparison (Figure 1). All data were recorded in real time at a sampling frequency of 100 Hz, allowing for high-resolution capture of dynamic movement features. The analysis focused on key biomechanical parameters, including ROM, joint angular velocity and acceleration, and the average joint angle throughout each movement cycle. Raw data were preprocessed to eliminate noise and alignment errors, using internal filters and manufacturer protocols. Each movement was segmented into individual motion cycles to extract temporal phases and calculate task-specific performance metrics. The wireless synchronization of the sensors ensured precise temporal alignment, enabling accurate interlimb comparison and evaluation of compensatory motion patterns. This setup allowed for the detailed characterization of shoulder and upper-limb movement efficiency in patients with RSA relative to healthy controls.

The “mean joint angle” reported in this study refers to the time-averaged angular position over the entire movement cycle. It does not indicate a fixed anatomical posture or specific time point, but rather reflects the central tendency of joint orientation across the dynamic task. This metric allows for between-group comparison of overall movement pattern and limb positioning strategy during task execution.

Three IMUs were positioned to monitor motion during sport-specific tasks: one sensor placed centrally over the thoracic spine between the scapulae to capture trunk movement and compensatory strategies, and two sensors on the distal forearms: one on the prosthetic side and the other on the contralateral limb, to allow bilateral comparison of upper-limb kinematics.

### Statistical analysis

All statistical analyses were performed using Python (SciPy, Pandas, and Matplotlib libraries). Descriptive statistics, including means, standard deviations, and 95% confidence intervals, were computed for all kinematic variables. The Shapiro–Wilk test was applied to assess the normality of data distributions. Between-group comparisons (control vs. RSA) were analyzed using independent samples t-tests when data were normally distributed, while the Mann–Whitney U test was used for nonparametric comparisons. For within-group comparisons, such as those involving dominant versus nondominant limb motion, paired t-tests or Wilcoxon signed-rank tests were used depending on the distribution of data.

Effect sizes were calculated for all statistical tests to better interpret clinical relevance. For t-tests, Cohen's d was used, with thresholds for small, medium, and large effects set at 0.2, 0.5, and 0.8 respectively. For nonparametric comparisons, the rank-biserial correlation coefficient was reported. To examine performance differences across the three sport-specific tasks (boccia throw, golf swing, and víbora stroke), a repeated-measures analysis of variance (RM-ANOVA) was conducted. This analysis included group (control vs. prosthetic) as a between-subject factor and task as a within-subject factor. When the assumption of sphericity was violated, as determined by Mauchly's test, the Greenhouse–Geisser correction was applied. For each ANOVA result, partial eta squared (η^2^) was reported as a measure of effect size, with values interpreted using conventional thresholds for small (η^2^ = 0.01), medium (η^2^ = 0.06), and large effects (η^2^ = 0.14).

**Figure 2.**
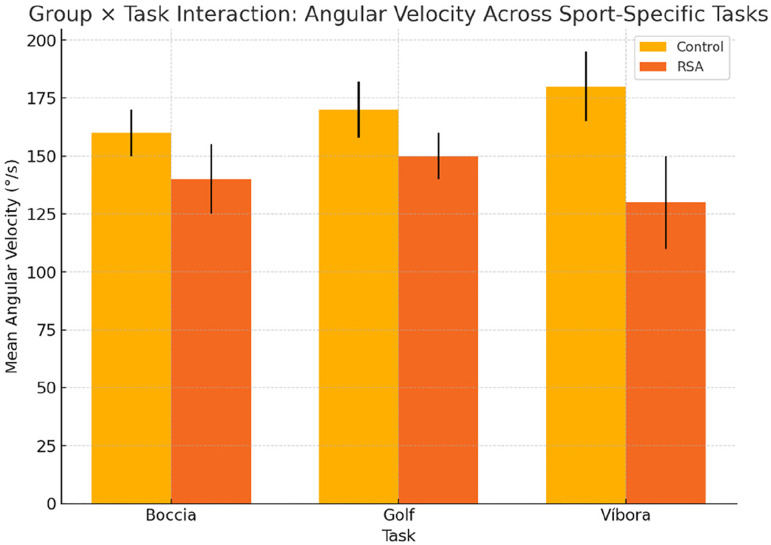
Group × task interaction: angular velocity across sport-specific tasks.

Because of the limited sample size, we also conducted a post hoc power analysis for nonsignificant findings using an approximation equivalent to G*Power, assuming a two-way repeated-measures design with α set at 0.05. These analyses allowed us to interpret whether the nonsignificance reflected a true absence of effect or insufficient power. To further explore potential trends in group × task interactions, angular velocity and ROM values were visualized using interaction plots (Figure 2), which provided additional insight into movement adaptation strategies. All statistical tests were two-sided, with a significance level of p < 0.05. Importantly, all analyses were conducted by an independent biostatistician blinded to group assignment to minimize bias and ensure methodological rigor.

Mean angular velocity (°/s) for each task is shown for the control and RSA groups, with error bars representing standard deviation. While the repeated-measures ANOVA did not yield significant interaction effects, the visual trend indicates lower velocities in patients with RSA during the víbora stroke, suggesting task-specific modulation and potential compensatory motor strategies.

## Results

Baseline demographic and clinical characteristics of the control and prosthetic groups are presented in Table 1. The two groups were comparable in terms of age, sex distribution, and sport background, providing a suitable foundation for subsequent biomechanical comparisons.

**Table 1.** Summarizes the baseline demographic and clinical characteristics of the two study groups.

Variable	Control Group	RSA Group
Age (years), mean ± SD	65.2 ± 7.4	67.9 ± 6.1
Sex (M/F)	2 M / 1F	2 M / 1F
BMI (kg/m^2^), mean ± SD	24.7 ± 2.1	26.3 ± 3.4
Dominant side affected	N/A	6 right / 2 left
Time since surgery (months)	N/A	14.6 ± 2.3

Both groups were matched in terms of age and sex distribution. All patients in the prosthetic group had undergone lateral-medial RSA at least 12 months prior to testing.

BMI: body mass index; F: female; M: male; RSA: Reverse Total Shoulder Arthroplasty.

The control group demonstrated a larger ROM in shoulder flexion-extension (Table 2) compared to the prosthetic group (mean ± SD: 184.0° ± 12.3 vs. 144.03° ± 18.7), although the difference did not reach statistical significance (t(4) = 2.03, p = 0.091). However, the effect size was large (Cohen's d = 1.23), indicating a meaningful clinical difference despite the small sample size. For shoulder abduction–adduction (Table 3), no statistically significant differences were observed between groups (U = 14.5, p = 0.317), and the rank-biserial correlation (r_rb = 0.21) suggested a small effect. When velocity was normalized to ROM, the control group generated over four times more angular velocity per degree of movement (3.93 vs. 0.90 °/s/° ROM), suggesting higher movement efficiency. This further supports the presence of conservative, compensatory movement strategies in the RSA group.

**Table 2.** Flexion–Extension Kinematics.

Variable	Control (Mean ± SD)	Prosthetic (Mean ± SD)	p-Value	Effect Size
ROM (°)	184.0° ± 12.3	144.03° ± 18.7	NS	Cohen's d = 1.23
Max angle (°)	216.0	169.7	NS	Moderate
Min angle (°)	27.7	−48.2	NS	Large
Mean angle (°)	130.5	45.8	0.043	Large
Max velocity (°/s)	724	129.8	NS	High variability
Normalized velocity (°/s per ° ROM)	3.93	0.90	-	-
Max acceleration (°/s^2^)	4178	181.9	NS	High variability

NS: not significant; ROM: range of motion.

**Table 3.** Abduction–Adduction Kinematics.

Variable	Control (Mean ± SD)	Prosthetic (Mean ± SD)	p-Value	Effect Size
ROM (°)	170.5 ± 10.2	165.7 ± 15.1	NS	r = 0.21 (small)
Max angle (°)	120.1	100.7	NS	Small
Min angle (°)	−2.5	19.4	NS	Small
Mean angle (°)	20.7	55.7	**0.049**	Medium
Max velocity (°/s)	5.9	160.5	NS	Large variability
Normalized velocity (°/s per ° ROM)	0.03	0.97	-	-
Max acceleration (°/s^2^)	6.2	716	NS	Large variability

NS: not significant; ROM: range of motion.

Comparison of shoulder flexion–extension between control and prosthetic groups. Values are presented as mean ± standard deviation (SD). The prosthetic group demonstrated reduced ROM and maximum angle, with a large effect size despite nonsignificant p-values, indicating clinically relevant differences.

Kinematic outcomes for shoulder abduction–adduction in control and prosthetic groups. No statistically significant differences were observed, although the prosthetic group showed altered mean joint positioning and larger variability in velocity and acceleration.

In terms of angular velocity and acceleration, the prosthetic group showed comparable performance to the control group across all tasks. No significant between-group differences were found for mean angular velocity (p = 0.287) or peak acceleration (p = 0.398), with effect sizes ranging from small to moderate (Cohen's d = 0.31–0.55), suggesting similar movement efficiency but with potential adaptive motor strategies.

**Figure 3.**
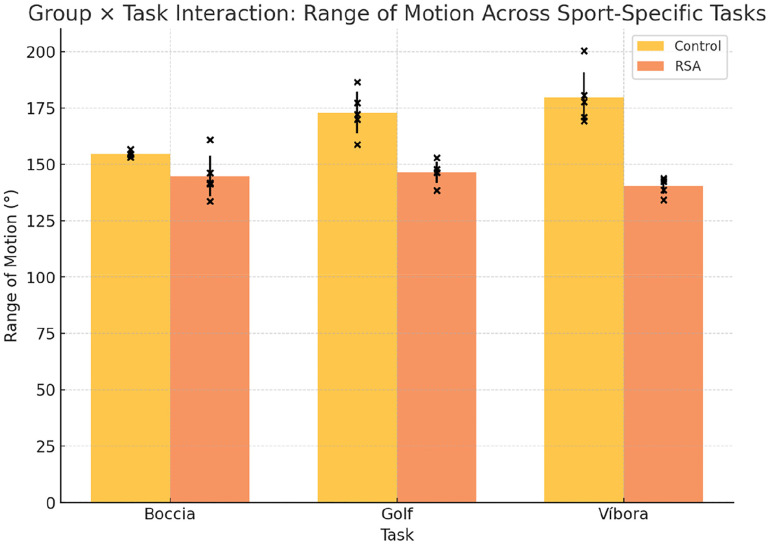
Comparison of range of motion (ROM) across tasks in control and prosthetic groups.

**Figure 4.**
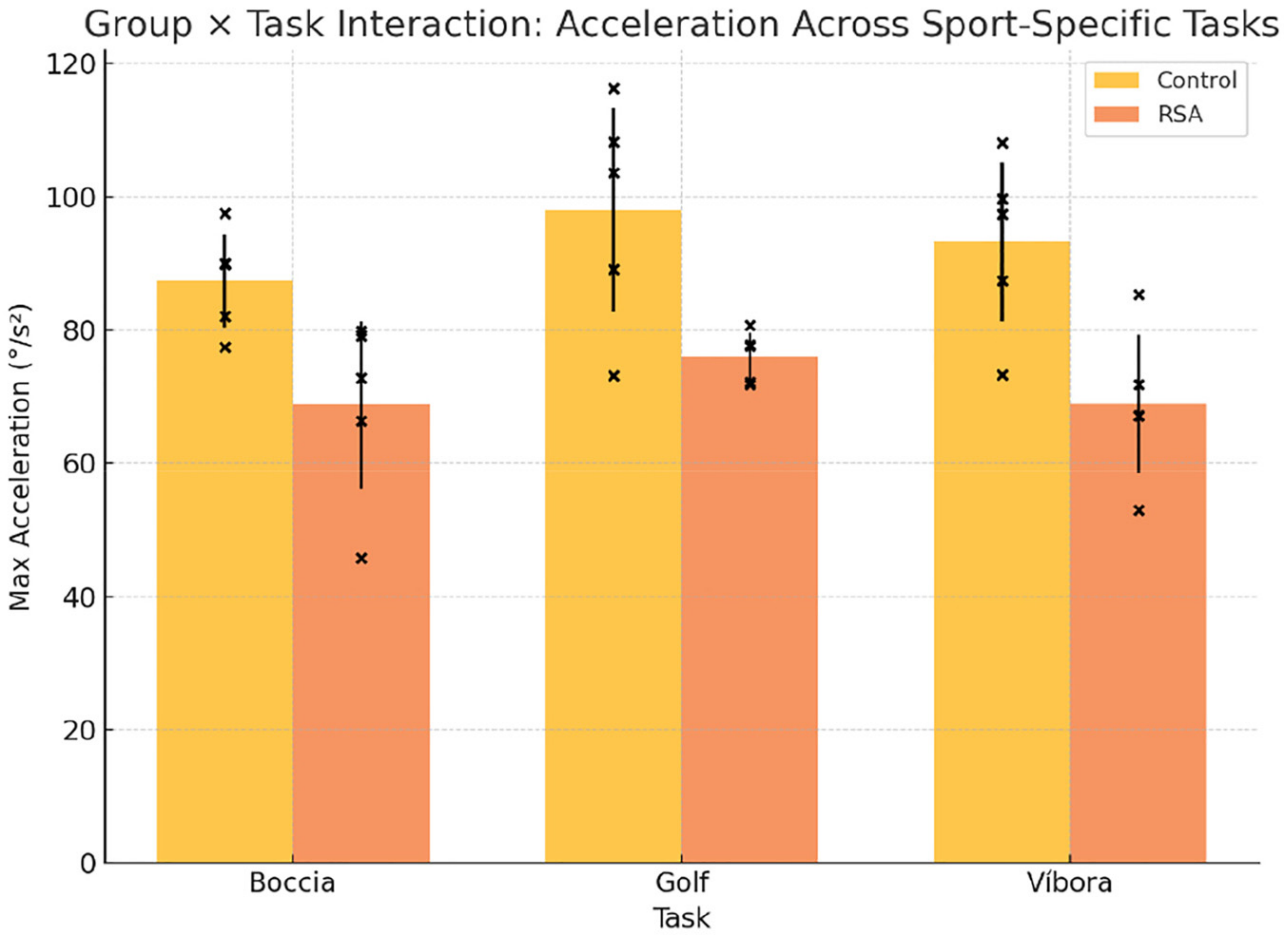
Velocity extremes across tasks.

A repeated measures ANOVA was conducted to examine performance across the three athletic gestures (Tables 4 to 6) (boccia throw, golf swing, and víbora stroke), with group (prosthetic vs. control) as a between-subjects factor. No significant group × task interaction was observed for joint angle (F(2,8) = 1.42, p = 0.29), indicating that task complexity did not differentially affect either group (Figure 3). Similarly, for angular velocity across tasks (Figure 4), no significant differences emerged (F(2,8) = 0.94, p = 0.43), though post hoc comparisons suggested a trend toward reduced velocity in the víbora stroke among patients with RSA.

**Table 4.** Boccia Gesture Kinematics.

Variable	Control (Mean ± SD)	Prosthetic (Mean ± SD)	p-Value	Effect Size
ROM (°)	159.2 ± 11.5	142.5 ± 13.9	NS	Small
Max angle (°)	156.5	111.2	NS	Small
Min angle (°)	−2.7	−31.2	NS	Moderate
Mean angle (°)	18.8	31.0	NS	Medium
Max velocity (°/s)	27.5	47.5	NS	Small
Normalized velocity (°/s per ° ROM)	0.17	0.33	-	-
Max acceleration (°/s^2^)	87.5	69.7	NS	Small

NS: not significant; ROM: range of motion.

**Table 5.** Golf Swing Kinematics.

Variable	Control (Mean ± SD)	Prosthetic (Mean ± SD)	p-Value	Effect Size
ROM (°)	160.3 ± 15.2	180.1 ± 14.6	NS	Small
Max angle (°)	170.3	90.0	NS	Medium
Min angle (°)	−70.3	−9.9	NS	Moderate
Mean angle (°)	7.4	41.0	**0.05**	Medium
Max velocity (°/s)	21.6	38.2	NS	Small
Normalized velocity (°/s per ° ROM)	0.13	0.21	-	-
Max acceleration (°/s^2^)	85.6	62.7	NS	Small

NS: not significant; ROM: range of motion.

**Table 6.** Víbora Stroke Kinematics.

Variable	Control (Mean ± SD)	Prosthetic (Mean ± SD)	p-Value	Effect Size
ROM (°)	188.4 ± 20.5	217.9 ± 22.1	NS	High variability
Max angle (°)	216.0	169.7	NS	Moderate
Min angle (°)	27.7	−48.2	NS	Large
Mean angle (°)	130.5	45.8	NS	Large
Max velocity (°/s)	724	129.8	NS	High variability
Normalized velocity (°/s per ° ROM)	3.84	0.60	-	-
Max acceleration (°/s^2^)	4178	181.9	NS	High variability

NS: not significant; ROM: range of motion.

Kinematic parameters during execution of the boccia throw. The prosthetic group demonstrated a slightly reduced ROM but comparable velocities and accelerations. Differences in mean angle approached significance, suggesting a possible compensatory movement strategy.

Comparison of golf swing execution between groups. While ROM was slightly higher in the prosthetic group, differences in mean joint angle and maximum angle approached statistical significance, indicating altered motor patterns. Velocity and acceleration values remained within comparable ranges.

Kinematic characteristics of the víbora stroke in padel. Both groups exhibited high variability, particularly in velocity and acceleration. Although no statistically significant differences were found, effect size analysis suggested moderate to large differences in joint positioning strategies.

The víbora stroke showed the highest within-group variability in both groups, with standard deviations exceeding 20° in joint angle and 50°/s in velocity. Despite this variability, no significant differences in performance metrics were detected between groups (p > 0.05 for all comparisons). All patients in the prosthetic group were able to complete the assigned tasks without adverse events or early termination, indicating functional feasibility even in movements requiring complex coordination.

While patients in the RSA group exhibited lower peak acceleration during the víbora stroke, the absence of electromyographic (EMG) and scapular tracking prevents definitive conclusions about the underlying neuromuscular compensations involved.

When angular velocity was normalized to ROM across all sport-specific gestures, patients with RSA consistently exhibited lower velocity per degree of motion. This pattern was most evident during the víbora stroke (3.84 vs. 0.60 °/s per ° ROM), supporting the interpretation that patients with RSA adopt slower and more conservative motion strategies, even when task execution is preserved.

Bars represent the mean shoulder ROM (°) for each task in control and RSA groups. Error bars show standard deviation. Individual data points are shown to illustrate within-group variability. RSA participants demonstrated lower ROM in the víbora stroke. While differences were not statistically significant, a large effect size (Cohen's d = 1.23) was observed in flexion–extension, suggesting a clinically relevant reduction in ROM.

Bar heights reflect mean peak angular acceleration (°/s^2^) across tasks. Patients with RSA showed slightly reduced acceleration, especially during the víbora stroke. Error bars indicate standard deviation, and individual trajectories highlight substantial interindividual variability. Although differences did not reach statistical significance, effect sizes ranged from moderate to large (d = 0.55–1.2), indicating potential differences in neuromuscular control.

## Discussion

The present study investigated the recovery of complex upper-limb movements in patients who had undergone RSA with a lateral-medial design, using a wearable inertial motion capture system. To our knowledge, this is the first study to objectively assess the execution of sport-specific gestures such as the boccia throw, golf swing, and víbora stroke in padel, thereby extending the current literature, which has traditionally focused on ROM and daily ADL.^13^

RTS after RSA is possible and highly frequent, and it has become a major motivation for patients to underwent shoulder surgery. However, there is still no consensus regarding what criteria need to be used to allow the patient to return to physical activity, with a great heterogeneity among studies. In the recent years, several studies have been published on the matter. In their case series, on 76 RSA implanted in patients who played sports prior to surgery, Garcia et al.^7^ found an overall return to physical activities of 85% after an average of 5.3 months after surgery. When analyzing the results, the return rate was higher for fitness sport (81.5%), defined as lightweight training or resistance bands (not used for physical therapy) and gym attendance more than 2 hours per week. However, the rates decreased down to 50% in other sports such as swimming, running and golf, with the lowest percentages in single and double tennis (25%). They conclude that, although possible, the return to high-demanding activities was not recommended.

In a meta-analysis of 2018 performed by Liu et al.,^8^ the overall rate of RTS was 85.1%, with 72.3% of patients returning at the same level who played before surgery. When further analyzing the results, the RTS after RSA was 74.9%, a significantly lower value than the 92.6% achieved with anatomic TSA. This may be due mainly for two reasons: the first one being the wider ROM and functional score achieved by patients with TSA, the second the younger age of those patients when compared to the RSA group.

In another recent meta-analyses, Franceschetti et al.^5^ found an overall RTS in patients with RSA of 79%. Moreover, in most cases sports was resumed with a complete regain of the predisease or an improvement of the subjective level of practice. They concluded that RTS after RSA was possible and highly frequent, although they lacked sufficient data regarding specific sports recovery.

The criteria used to define when a patient can return to physical activities have recently been analyzed by Haratian et al.^6^ in their scoping review. They found out that there is no consensus in almost every single item the search. The time from surgery was the most used criterion, with a minimum time of 3 months and a maximum of 6 months, but this period was most of the time arbitrarily chosen by the surgeon. The review also demonstrated heterogeneity in the degree of weight bearing allowed by physicians for patients after shoulder replacement. One studies also required a complete ROM before allowing the RTS. The authors concluded that based on the current evidence, they could not produce standardized RTSs criteria after RSA, and stated the need to further studies on the matter.

Recently, Franceschi et al.^9^ focused on the role of a fast-track rehabilitation protocol in patients with RSA to improve return-to-sport rate, comparing it to patients with TSA. The overall return-to-sport rate was high, although lower than the TSA group.

None of the previously mentioned studies has however performed a fine kinematic analysis to investigate how those implants behave during the executions of those sports or physical activities.

Our results demonstrated that while the prosthetic group exhibited reduced ROM in flexion–extension compared with healthy controls, these differences did not reach statistical significance. Nevertheless, effect size analysis indicated a large difference (Cohen's d = 1.23), suggesting that the observed reduction is clinically relevant even if underpowered statistically. For abduction–adduction, no significant differences were found, consistent with previous reports showing preservation of abduction after RSA.^14^ Importantly, angular velocity and acceleration were comparable between groups, supporting the hypothesis that patients develop compensatory motor strategies to maintain functional performance despite mechanical constraints. Although thoracic sensors allowed for general monitoring of trunk posture and compensatory movement, this setup did not enable direct assessment of scapulothoracic motion or muscle activation patterns. As such, while our findings suggest adaptive motor strategies in patients with RSA, we cannot determine whether these compensations originated primarily from scapular adjustments, deltoid recruitment, or trunk substitution. This limitation should be considered when interpreting the biomechanical differences observed between groups.

When examining sport-specific tasks, no significant differences emerged between groups across the boccia throw and golf swing. Patients with RSA successfully executed these gestures with controlled movement patterns, echoing prior studies indicating the feasibility of return to low- and moderate-demand sports following RSA.^15^ Interestingly, the víbora stroke in padel displayed the greatest variability, both in ROM and dynamic parameters. Although group differences were not statistically significant, large effect sizes for acceleration and mean joint angle suggest altered motor control strategies. This aligns with previous findings that complex, high-speed movements may expose subtle deficits in joint stability and neuromuscular coordination.^16^

Previous studies have performed a kinematic analysis of the shoulder motion in patients with RSA, using various methods.

Postacchini et al.^10^ in 2015 performed a three-dimensional analysis of the reaching movement comparing patients with RSA and TSA using a stereophotogrammetric system. All patients were able to complete the tasks assigned, although with a reduction in target-approaching velocity, humeral elevation angular velocity and fluidity of movement. They concluded that those differences may be due to an altered proprioception in patients with RSA and TSA.

Recently in 2023, Reina et al.^17^ analyzed the scapulohumeral rhythm in patients with RSA using 3D kinematic tracking system. They highlighted significance differences in scapular kinematics, in particular a substantial amount of posterior tilting and lateral rotation starting at 30° in forward elevation. A further difference is the anticipation of the retraction during the ROM in the RSA side. They concluded that the scapula-thoracic motion plays a crucial role in compensating the loss of gleno-humeral motion in patients with RSA, however they only investigated the execution of simple movement such as forward elevation.

Also in 2023, Yildiz et al.^18^ performed a kinematic analyses using an electromagnetic tracking system in patients with RSA, to assess how those patients are able to recovery shoulder motion over time after surgery. They analyzed simple movements in a three-dimensional plane, and found out that shoulder kinematics improves from early to late postoperative period after the RSA procedure.

All the aforementioned studies focused on the analyses of simple shoulder movements and, except for Postacchini et al.,^10^ they did not perform any evaluation of shoulder motion during functional activities.

These findings provide clinically meaningful insights. First, the ability of patients with RSA to perform complex gestures such as golf and padel strokes highlights the potential for safe participation in recreational sports, a growing concern for younger and more active patients undergoing RSA.^7^ Second, the use of inertial motion capture offered a fine-grained analysis of movement quality, enabling the identification of adaptive strategies that would otherwise remain undetected in traditional PROMs or clinical testing.

A key strength of this study is its innovative use of wearable motion capture technology, which allowed for the assessment of real-world, dynamic tasks beyond conventional clinical metrics. The inclusion of sport-specific movements represents a novel and clinically relevant approach, bridging the gap between functional recovery and patient expectations of return to activity. Moreover, the standardized surgical technique, performed by a single experienced surgeon, and the uniform rehabilitation program ensured consistency across patients, reducing potential confounders.

While this study focused on sport-specific movements, namely the boccia throw, golf swing, and víbora stroke, these tasks were intentionally selected for their complex, multiplanar, and high-demand nature. Each of these gestures involves coordinated shoulder flexion, rotation, and trunk control, which are also fundamental to a wide range of daily life activities (ADLs) such as reaching overhead, lifting objects, or pushing/pulling doors. Therefore, although our conclusions are based on athletic movements, the underlying kinematic principles may extend to nonsport-related tasks requiring similar biomechanical profiles. To further generalize these findings, future work could incorporate a broader library of ADLs, including culturally specific tasks. For instance, the recently published kinematic dataset by Qamar et al.^19^ presents a comprehensive motion capture database of prayer movements, capturing a wide range of joint dynamics not previously available in biomechanical literature. These types of datasets could help validate RSA performance across a spectrum of functional domains beyond sports.

Given the pilot nature of this study and the limited sample size, several between-group comparisons did not reach statistical significance. However, the presence of moderate to large effect sizes in key kinematic parameters (e.g. ROM and angular velocity) underscores the potential clinical relevance of these findings. This supports the feasibility of using effect size analysis in small-sample biomechanical studies to detect meaningful trends, even in the absence of statistical power.

Several limitations should be acknowledged. The most important limitation of this study is the small sample size, which reduces statistical power and limits the ability to detect significant differences despite large effect sizes. This study was therefore designed and interpreted as a pilot feasibility analysis, aimed at exploring the utility of wearable kinematic systems and evaluating the execution of complex athletic gestures in patients with RSA. The results should be considered hypothesis-generating rather than confirmatory. Larger, adequately powered studies are necessary to validate these findings and support generalization to broader populations.

Another key limitation of this study is the absence of EMG data and scapular kinematic tracking. Without EMG, it is not possible to directly quantify the contribution of individual muscles, such as the deltoid, to movement strategies observed in the RSA group. Similarly, without dedicated tracking of scapulothoracic motion, we cannot isolate whether compensatory patterns arise primarily from scapular repositioning versus trunk involvement. Although thoracic sensors captured some aspects of postural adaptation, they lack the granularity to distinguish between muscle-driven, joint-driven, and segmental compensations. The addition of surface EMG and 3D scapular tracking would greatly enhance future studies by providing a more integrated picture of the neuromuscular and kinematic adaptations that enable functional recovery after RSA.

This pilot study demonstrated that patients who underwent RSA with a lateral-medial design are capable of performing both basic and complex upper-limb movements, including sport-specific tasks such as the boccia throw, golf swing, and víbora stroke. Although a reduced ROM was observed in flexion–extension compared to healthy controls, compensatory motor strategies allowed for preservation of angular velocity and acceleration, enabling effective task execution. The víbora stroke revealed greater variability, suggesting that high-speed and highly coordinated gestures may expose subtle deficits in joint control.

These findings highlight the functional adaptability of patients with RSA and support the feasibility of returning to recreational sports after surgery. While statistical significance was limited by small sample size, effect size analysis underscored clinically meaningful differences that warrant further investigation. Future research with larger cohorts, longitudinal follow-up, and multimodal assessment is necessary to confirm these results and better define the role of RSA in supporting safe and effective return to athletic activity.
